# Foundational issues, analysis and geometry in continuum mechanics: introduction

**DOI:** 10.1098/rsta.2022.0376

**Published:** 2023-12-25

**Authors:** Paolo Maria Mariano, Anja Schlömerkemper

**Affiliations:** ^1^ DICEA, University of Florence, via Santa Marta 3, 50139 Firenze Italy; ^2^ University of Würzburg, Institute of Mathematics, Emil-Fischer-Str. 40, D-97074 Würzburg, Germany

**Keywords:** continuum mechanics, mathematical analysis, geometry

Continuum mechanics—we can say—is a class of field theories, based on causality, which describe bodies extended in space and do not account directly for the quantum structure of the matter. With words of an imaginary theoretic physician involved in the analysis of atomic and subatomic behaviour, we could say in a sense that continuum mechanics emerges at long wavelength approximation. Alternatively, looking from the kinetic theory of gases, we might as well say that continuum mechanics comes into play when the number of molecules tends to infinity.

As a class of classical (in the sense of non-quantized) field theories, continuum mechanics has been so far an attractive playground for researchers working on calculus of variations, differential geometry, numerical analysis and partial differential equations. They found settings in which they could test their abstract methods under constraints suggested by the physics—*per se* models are *representations* of physical phenomena only possibly corroborated by experiments or falsified by them. In turn, physical evidence may suggest the development of abstract tools.

A particularly significant case is the increase of interest for the analysis of polyconvex energies for simple elastic bodies with respect to the deformation gradient F, which has been determined by the physical incompatibility between energy convexity with respect to F and the requirement of objectivity for the energy itself [1–5].

Variational methods have been also fruitful in analysing dissipative processes not involving inertia: it is assumed that between discrete instants the path followed in state space minimizes energy and maximizes dissipation, a line of thought resting on E. De Giorgi’s minimal path technique [6–10]. A crucial problem is evaluating a limit as the length of time goes to zero, in order to reach the continuum time setting.

Admissibility conditions dictated by thermodynamics for shock-wave-type solutions of hyperbolic partial differential equations (the so-called entropy criteria) are another evident example [11].

Non-trivial analytical questions emerge in various sub-fields of continuum mechanics and are still open: a prominent (and in a sense paradigmatic) example is the evaluation of global-in-time regularity for solutions to the three-dimensional Navier–Stokes equations describing the motion of incompressible viscous simple fluids; in other words, the question is whether a smooth flow and a smooth scalar pressure can exhibit spontaneous formation of singularities [12–14]. Besides its obvious analytical interest for the theory of regularity (which has been developed in an appropriate sense also for classes of stochastic partial differential equations), such a question is expected to be crucial in the understanding of turbulence [15]. In turn, a related basic question emerges: whether the assumptions that give rise to the Navier–Stokes equations constitute an appropriate setting for turbulence; in other words, we may ask whether the scheme of simple (incompressible and viscous) fluids is appropriate to describe the transition from laminar to turbulent flows. Physics shows us such a transition; so, under question is not its existence, rather the way we describe it.

This type of questions motivates foundational analyses of models and/or theories (*per se* classes of interconnected models). A question remains in fact vigilant every time we construct a model: What are we actually doing?

Clarifying what is at the ground of a model or, better, a theory has even practical effects, besides its indisputable theoretical interest: it addresses, in fact, analytical and computational choices; it allows one to better understand the nature of approximations, avoiding as much as possible their ad hoc introduction only for the sake of convenience; it clarifies the nature of boundary conditions, distinguishing those physically admissible from those that are not so. In short, besides their clarifying nature, such a type of analysis may have not only have an inspiring role for mechanics and pure mathematics but also an inspiriting role.

Starting from the middle of the twentieth century, C. A. Truesdell III promoted, with his school, a foundational program in continuum mechanics and thermodynamics, which started with the fundamental work by W. Noll [16], primarily addressed towards the analysis of those bodies for which we find it appropriate to represent their morphology only in terms of a region that they may occupy in the physical space (we can call them *Cauchy’s bodies* in short, because for them the representation of contact—in the sense of first-neighbour—interactions rests only on Cauchy’s stress tensor).

Two articles for the Handbuch der Physik, two treatises indeed [17,18], have been landmarks in Truesdell’s program but they did not exhaust that program. Subsequent work on the foundations of continuum thermodynamics produced essential results (see the treatises [19–21]). Further on, considering time-varying reference configurations as a way to picture evolution of body sub-regions, relative to the rest, allowed analysis of defect evolution and interfaces in phase transitions, governed by independent balances of configurational actions (it seems that the adjective has been introduced by F. R. N. Nabarro [22–26]). When reduced to conservative settings, configurational balances coincide with those derived via horizontal variations (those involving the domain variation) of an action functional. The independence of the configurational balances from the balance of standard forces in the presence of irreversible evolution of defects confirms in a non-conservative setting what was already known in calculus of variations since W. R. Hamilton’s work, namely the independence of equations derived via horizontal variations from the Euler–Lagrange equations (for this historical aspect see pp. 152–153 in vol. I of the treatise [27]).

Also, and independently, considering concentrated forces and the associated conceptual difficulty in determining on which part of the body they have to be assigned, or whether they have to be shared between two sub-bodies when we ideally cut the body itself with Cauchy’s procedure motivating tension [28], implied a re-discussion of Noll’s algebra of bodies (an intrinsic abstract way to define interactions [29,30]) with the consequent need of considering measure-valued stress fields [31] (see also [32,33]). They also emerge when we take an energy that is polyconvex with respect to F and impose that the pertinent stress field might take values only in a bounded convex set (the approach requires introduction in large strain regime of a functional that can be considered in a precise sense as a counterpart of what is called a *complementary* energy in small strain regime) [34]. Conceptually, these aspects fall, however, within the traditional paradigm detailed in the two articles collected in the Handbuch der Physik already mentioned.

A sliding in such a paradigm emerged when, progressively, the analysis of condensed matter suggested to enrich the description of body morphology and its changes beyond the selection of a fit region in the physical space, in order to account for across-scale phenomena driven by interactions not completely representable in terms of standard Cauchy’s stress, or its versions obtained by pulling back in the reference configuration of one or both components. Indeed, the seminal idea dates back to the 1909 work by E. and F. Cosserat, who considered every material element not as a mere point in space, rather as a small rigid body able to rotate independently of its neighbours [35]. This approach was left aside for circumstantial reasons until 1958 when Ericksen & Truesdell [36] raised attention over it as a fruitful view for building up direct models for shells, rods, and their like [37–41] (see also related works [42,43]). Starting from 1960, Ericksen himself adopted that ‘philosophy’ of considering every material element as a system rather than a point to represent the dynamics of liquid crystals, above all those showing nematic order [44–47]. Essentially, the idea was to couple strain with phase-fields adopted in condensed matter physics (see the extended critical review [48]) to describe microstructures. The approach generated a rich crop of proposals for the description of various physical circumstances in which microstructural events have gross-scale effects (porous bodies, liquids with bubbles, ferroelectrics, magnetostrictive materials, etc.). It was thus evident that a view considering the construction of a mechanical model to be reduced only to a selection of special constitutive relations is at least restrictive. The construction of a mechanical model starts, in fact, from the way we decide to describe the morphology of a body and its changes, which influences the representation of actions, and thus affects the choice of constitutive structures. A foundational problem concerning the wide taxonomy of specific models was thus to find whether they could be considered as offspring of a common ground. A proposal in this sense by Germain dates back to 1973 [49]: he considered descriptors of the material morphology in a linear space and the principle of virtual work as a guiding rule. In so doing, he assumed *a priori* the weak form of balance equations, which presumes the representation of microstructural interactions; then, he did not use *per se* such weak form (namely for determining existence of weak solutions to boundary value problems or to develop pertinent numerical schemes), accepting the regularity implying local balance equations.

In a 1989 book [50], Capriz summarized and pushed forward aspects of his previous work on this matter (e.g. [50–53]) and proposed a concrete unifying framework in which descriptors of material morphology are taken in a generic finite-dimensional differentiable manifold (encompassing in this way models left out by the previous attempt unless geometric constraints are inserted, a case not treated in that attempt) and postulated balance equations in their local form: they include the balance of microstructural interactions and its link with the balance of couples. Foundational questions emerged: Are the balance of microstructural interactions to be considered as first principles or can they be deduced from a more fundamental first principle (exactly as is for the balances of standard forces and couples, according to a 1959 result by Noll [54])? Before this aspect: How can we deduce an intrinsic representation of microstructural interactions maintaining as general as possible the underlying manifold structure? But also analytical problems emerged, for example: Under what conditions do minimizers of appropriate energies for a generic complex body exist? Answers to these questions have been provided along the years (e.g. [55–62]); at times they have been decisive; often they open paths leading to further foundational issues that involve, in turn, algebraic, analytical and geometrical non-trivial questions to various degrees.

However, it is not our purpose to propose here a history of ideas in mechanics^1^; rather, we just aim at underlying that continuum mechanics is a dynamic variegate field at various conceptual levels, a land that can flourish even in unexpected places.

To furnish a tangible perception of this view, in the present ‘Theme Issue’, we collected research papers proposing foundational analyses, specific models, existential and representation results; they also constitute examples of how algebraic, analytical, geometrical and foundational issues may fruitfully interact in continuum mechanics.

Specifically, Bachman *et *al*.* [63] consider a one-dimensional stress-rate type model, which is reminiscent of Truesdell’s hypoelasticity; it implies a balance of forces expressed by a peculiar partial differential equation with homogeneous Neumann’s boundary conditions; its initial data are selected in a Gevrey’s class with 3/2 regularity. Under these conditions, local existence of solutions to the pertinent linearization are determined around certain steady-state states.

Brazda *et *al*.* [64] determine existence of minimizers for energies of two-phase elastic solids endowed with sharp interfaces described by curvature varifolds (which are vector-valued non-negative Radon measures supported by a rectifiable set and defined over a corresponding Grassmanian); the choice, which follows Giaquinta–Mariano–Modica–Mucci’s approach to crack surface energy, implies a penalization due to bending effects described by the extended notion of curvature that is pertinent to varifolds.

Du & Man [65] consider possible aggregates of crystallites in a crystal class with symmetry defined by any of the 21 improper points groups; in these circumstances, they provide a tensor-based Fourier expansion of the orientation distribution function describing the crystallographic texture, a function now defined over O(3) rather than on its Lie subgroup SO(3), commonly involved after the 1965 independent analyses of the orientation distribution function by Bunge and Roe; concrete examples enlighten the general proposal.

Duda *et *al*.* [66] develop an analysis having as a final target the mechanochemical coupling; they first consider a scenario in which a single constituent body experiences adsorption/desorption through its boundary; in this setting, they develop a pertinent model that is consistent with thermodynamic restrictions, analyse stability of steady states in a special geometry and establish conditions for the occurrence of instabilities due to surface phenomena; then, they arrive at the mechanochemical coupling, including the effects of strain.

Goldshtein *et *al*.* [67] furnish a new view on the elastic-anelastic multiplicative decomposition of F based on Segev’s idea of embodiment of a protobody (a collection B of material elements not necessarily endowed with manifold structure), namely an equivalence class of configurations for a protobody not required to be determined by an injective mapping. When restricted to crystals, the treatment involves tangent bundle morphims; more in general it may describe phenomena such as fracture and annihilation or reformation of material points.

Mariano [68] discusses the role of the second law of thermodynamics, written in terms of a mechanical dissipation inequality involving a power of actions that accounts for time-varying reference configurations. At variance of the traditional use, he shows that the representation of contact actions and the local balance can be deduced from the second law in addition to constitutive restrictions once we accept a *covariance principle* stating that if a given observer evaluates a process to be dissipative, any other observer should make the same evaluation.

Markenscoff [69] looks also at the Noether theorem and its pertinence to the analysis via horizontal variations of dislocation motion inside crystals; she considers the asymptotic expression of the displacement gradient around a dislocation at the order ε (a parameter determining the infinitesimal variation of dislocation placement); then, she proves that there is a loss of quasi-momentum (the pull-back of momentum into the reference configuration) between the scales ε and $\varepsilon^{2}$.

Mucci & Saracco [70] tackle a very classical problem dating back to Euler: equilibrium of the elastica; however, instead of considering only smooth curves, more generally they look at rectifiable shapes; to them they attribute energies depending on the curvature p-power, p>1; the resulting weak notion of bending energy, obtained by relaxation through inscribed polygonal, is finite in the presence of Sobolev’s regularity. They prove that a rectifiable curve has finite p-curvature for some p>1 if and only if the pertinent ‘curve’ of oriented unit tangent vectors is a Sobolev map with the same exponent p.

Pradhan & Yavari [71] consider a growing body (positive or negative growth) as a three-dimensional Riemannian manifold with *a priori* unknown metric defined by solving an appropriate boundary value problem; the scheme involves time-of-attachment and time-of-detachment maps describing, respectively, addition and loss of material elements. In this setting and as a special example, they compute stretch and stress in a thick hollow circular cylinder made of an incompressible isotropic material under a finite time-dependent extension and ablation/accretion processes.

Ruggeri [72] looks at Eckart’s equations (they are a relativistic version of Navier–Stokes–Fourier balances) from the viewpoint of kinetic theory, in particular moving from his theory of ‘rational extended thermodynamics’, and refers to a compressible and possibly dense gas; he eventually shows that any causal system of divergence type that satisfies the entropy principle with convex entropy converges to the Eckart system in the Maxwellian iteration (introduced by Ickeberry and Truesdell), even when no information at macroscopic scale are available from kinetic theory.

## Data Availability

This article has no additional data.

## Footnotes

1 References listed at the end are useful as a departure for further exploration of the pertinent literature.

## References

[1] Ball JM. 1976/1977 Convexity conditions and existence theorems in nonlinear elasticity. Arch. Ration. Mech. Anal. **63**, 337-403. (10.1007/BF00279992)

[2] Ball JM. 2002 Some open problems in elasticity. In *Geometry, mechanics and dynamics* (eds P Newton, P Holmes, A Weinstein), pp. 3–59. New York, NY: Springer.

[3] Giaquinta M, Modica G, Souček J. 1989 Cartesian currents, weak diffeomorphisms and existence theorems in nonlinear elasticity. Arch. Ration. Mech. Anal. **106**, 97-159. Erratum and addendum. *Arch. Rational Mech. Anal.*, (1990) **109**, 385–392. (10.1007/BF00251429)

[4] Giaquinta M, Modica G, Souček J. 1998 Cartesian currents in the calculus of variations, voll. I and II. Berlin, Germany: Springer.

[5] Müller S, Tang Q, Yan BS. 1994 On a new class of elastic deformations not allowing for cavitation. Ann. Inst. H. Poincaré Anal. Non Linéaire **11**, 217-243. (10.1016/s0294-1449(16)30193-7)

[6] De Giorgi E. 1993 New problems on minimizing movements. In *Ennio De Giorgi - Selected Papers* (eds L Ambrosio, G Dal Maso, M Forti, M Miranda, S Spagnolo), pp. 699–713. Springer, 2006.

[7] Francfort GA, Mielke A. 2006 Existence results for a class of rate-independent material models with non-convex elastic energies. J. Reine Angew. Math. **595**, 55-91. (10.1515/CRELLE.2006.044)

[8] Mielke A. 2002 Finite elastoplasticity, Lie groups and geodesics on SL(d). In *Geometry, dynamics and mechanics* (eds PK Newton, A Weinstein, PJ Holmes), pp. 61–90. New York, NY: Springer.

[9] Mielke A. 2004 Existence of minimizers in incremental elasto-plasticity with finite strains. SIAM J. Math. Anal. **36**, 384-404. (10.1137/S0036141003429906)

[10] Mielke A, Roubíc̆ek T. 2015 Rate-independent systems. Berlin, Germany: Springer.

[11] Dafermos C. 2005 Hyperbolic conservation laws in continuum physics. New York, NY: Springer.

[12] Robinson JC, Rodrigo JL, Sadowski W. 2016 The three-dimensional Navier–Stokes equations: classical theory. Cambridge, UK: Cambridge University Press.

[13] Seregin G. 2015 Lecture notes on regularity theory for Navier-Stokes equations. Singapore: Word Scientific.

[14] Constantin P, Foias C. 1988 Navier-Stokes equations. Chicago, IL: The University of Chicago Press.

[15] Temam R. 2014 Turbulence and Navier Stokes equations. Berlin, Germany: Springer.

[16] Noll W. 1958 A mathematical theory of the mechanical behavior of continuous media. Arch. Ration. Mech. Anal. **2**, 197-226. (10.1007/BF00277929)

[17] Truesdell CA, Noll W. 1965 *The non-linear field theories of mechanics*. In *Handbuch der Physik*, Band III/3, pp. 1–602. Berlin, Germany: Springer.

[18] Truesdell CA, Toupin RA. 1960 *Classical field theories of mechanics*. In *Handbuch der physics*, pp. 226–793. Berlin, Germany: Springer.

[19] Ruggeri T, Sugiyama M. 2015 Rational extended thermodynamics beyond the monatomic gas. Berlin, Germany: Springer.

[20] Šilhavý M. 1997 The mechanics and thermodynamics of continuous media. Berlin, Germany: Springer.

[21] Truesdell CA. 1984 Rational thermodynamics. Berlin, Germany: Springer.

[22] Ericksen JL. 1998 On nonlinear elasticity theory of crystal defects. Int. J. Plast. **14**, 9-24. (10.1016/S0749-6419(97)00037-5)

[23] Eshelby JD. 1975 The elastic energy momentum tensor. J. Elast. **5**, 321-335. (10.1007/BF00126994)

[24] Gurtin ME. 1995 The nature of configurational forces. Arch. Ration. Mech. Anal. **131**, 67-100. (10.1007/BF00386071)

[25] Gurtin ME. 2000 Configurational forces as basic concepts of continuum physics. New York, NY: Springer.

[26] Maugin GA. 1995 Material forces: concepts and applications. Appl. Mech. Rev. **48**, 213-245. (10.1115/1.3005101)

[27] Giaquinta M, Hildebrandt S 1996 Calculus of variations, vol. I, The Lagrangian Formalism, vol. II, The Hamiltonian Formalism. Berlin, Germany: Springer.

[28] Podio-Guidugli P. 2004 Examples of concentrated contact interactions in simple bodies. J. Elast. **75**, 167-186. (10.1007/s10659-005-3029-8)

[29] Noll W. 1973 Lectures on the foundations of continuum mechanics and thermodynamics. Arch. Ration. Mech. Anal. **52**, 62-69. (10.1007/BF00249093)

[30] Truesdell CA. 1977 A first course in rational continuum mechanics. New York, NY: Academic Press.

[31] Schuricht F. 2007 A new mathematical foundation for contact interactions in continuum physics. Arch. Ration. Mech. Anal. **184**, 495-551. (10.1007/s00205-006-0032-6)

[32] Šilhavý M. 1991 Cauchy’s stress theorem and tensor fields with divergence measure in $L^{p}$. Arch. Ration. Mech. Anal. **116**, 223-255. (10.1007/BF00375122)

[33] Šilhavý M. 2005 Divergence measure fields and Cauchy’s stress theorem. Rendiconti Seminario Mat. Univ. Padova **113**, 15-45.

[34] Giaquinta M, Mariano PM, Modica G. 2015 Stress constraints in simple bodies undergoing large strain: a variational approach. Proc. R. Soc. Edinb. **145A**, 1-32. (10.1017/S0308210515000384)

[35] Cosserat E, Cosserat F. 1909 Sur la theorie des corps deformables. Paris, France: Dunod.

[36] Ericksen JL, Truesdell CA. 1958 Exact theory of stress and strain in rods and shells. Arch. Ration. Mech. Anal. **1**, 295-323. (10.1007/BF00298012)

[37] Antman SS 1972 The theory of rods. Handbuch der Physik VIa/2. Berlin, Germany: Springer, pp. 641-703.

[38] Antman SS. 1995 Nonlinear problems of elasticity. Berlin, Germany: Springer.

[39] Bhattacharya K, James RD. 1999 A theory of thin films of martensitic materials with applications to microactuators. J. Mech. Phys. Sol. **47**, 531-576. (10.1016/S0022-5096(98)00043-X)

[40] Friesecke G, James RD. 2000 A scheme for the passage from atomic to continuum theory of thin films, nanotubes and nanorods. J. Mech. Phys. Solids **48**, 1519-1540. (10.1016/S0022-5096(99)00091-5)

[41] Simo JC, Marsden JE, Krishnaprasad PS. 1988 The Hamiltonian structure of non- linear elasticity: the material and convective representations of solids, rods and plates. Arch. Ration. Mech. Anal. **104**, 125-183. (10.1007/BF00251673)

[42] Friesecke G, James RD, Müller S. 2002 A theorem on geometric rigidity and the derivation of nonlinear plate theory from three-dimensional elasticity. Commun. Pure Appl. Math. **55**,1461-1506. (10.1002/cpa.10048)

[43] Friesecke G, James RD, Müller S. 2006 A hierarchy of plate models derived from nonlinear elasticity by gamma-convergence. Arch. Ration. Mech. Anal. **180**, 183-236. (10.1007/s00205-005-0400-7)

[44] Ericksen JL. 1960 Theory of anisotropic fluids. Trans. Soc. Rheol. **4**, 29-39. (10.1122/1.548864)

[45] Ericksen JL. 1961 Conservation laws for liquid crystals. Trans. Soc. Rheol. **5**, 23-34. (10.1122/1.548883)

[46] Ericksen JL. 1962 Hydrostatic theory of liquid crystals. Arch. Ration. Mech. Anal. **9**, 371-378. (10.1007/BF00253358)

[47] Ericksen JL. 1991 Liquid crystals with variable degree of orientation. Arch. Ration. Mech. Anal. **113**, 97-120. (10.1007/BF00380413)

[48] Mermin ND. 1979 The topological theory of defects in ordered media. Rev. Mod. Phys. **51**, 591-648. (10.1103/RevModPhys.51.591)

[49] Germain P. 1973 The method of virtual power in continuum mechanics. Part 2: microstructure. SIAM J. Appl. Math. **25**, 556-575. (10.1137/0125053)

[50] Capriz G. 1989 Continua with microstructure. Berlin, Germany: Springer.

[51] Capriz G, Podio-Guidugli P. 1976 Discrete and continuous bodies with affine structure. Ann. Mat. Pura Appl.: (4) **111**, 195-211. (10.1007/BF02411819)

[52] Capriz G, Podio-Guidugli P. 1983 Structured continua from a Lagrangian point of view. Ann. Mat. Pura Appl. **135**, 1-25. (10.1007/BF01781060)

[53] Capriz G. 1985 Continua with latent microstructure. Arch. Ration. Mech. Anal. **90**, 43-56. (10.1007/BF00281586)

[54] Noll W. 1963 La Mécanique classique, basée sur une axiome d’objectivité, pp. 47–56 of *La Méthode Axiomatique dans les Mécaniques Classiques et Nouvelles* (Colloque International, Paris, 1959), Gauthier-Villars, Paris.

[55] Gay-Balmaz G, Ratiu T. 2009 The geometric structure of complex fluids. Adv. Appl. Math. **42**, 176-275. (10.1016/j.aam.2008.06.002)

[56] Holm DD. 2002 Euler-Poincaré dynamics of perfect complex fluids. In *Geometry, dynamics and mechanics* (eds PK Newton, A Weinstein, PJ Holmes), pp. 61–90. New York, NY: Springer.

[57] Mariano PM. 2002 Multifield theories in mechanics of solids. Adv. Appl. Mech. **38**, 1-93. (10.1016/S0065-2156(02)80102-8)

[58] Mariano PM. 2014 Mechanics of material mutations. Adv. Appl. Mech. **47**, 1-92. (10.1016/B978-0-12-800130-1.00001-1)

[59] Mariano PM, Modica G. 2009 Ground states in complex bodies. ESAIM - Contr. Optim. Calc. Var. **15**, 377-402. (10.1051/cocv:2008036)

[60] Segev R. 1994 A geometrical framework for the static of materials with microstructure. Math. Mod. Methods Appl. Sci. **4**, 871-897. (10.1142/S0218202594000480)

[61] Segev R. 2016 Continuum mechanics, stresses, currents and electrodynamics. Phil. Trans. R. Soc. A **374**, art. n. 20150174. (10.1098/rsta.2015.0174) 27002071

[62] Capriz G, Giovine P. 1997 On microstructural inertia. Math. Mod. Methods Appl. Sci. **7**, 211-216. (10.1142/S021820259700013X)

[63] Bachmann L, De Anna F, Schlömerkemper A, Şengül Y. 2023 Existence of solutions for stress-rate type strain-limiting viscoelasticity in Gevrey spaces. Phil. Trans. R. Soc. A **381**, 20220374. (10.1098/rsta.2022.0374) 37926215 PMC10645087

[64] Brazda K, Kružík M, Rupp F, Stefanelli U. 2023 Curvature-dependent Eulerian interfaces in elastic solids. Phil. Trans. R. Soc. A **381**, 20220366. (10.1098/rsta.2022.0366) 37926208

[65] Du W, Man C-S. 2023 Tensorial Fourier expansion of orientation distribution function defined on the orthogonal group O(3). Phil. Trans. R. Soc. A **381**, 20220368. (10.1098/rsta.2022.0368) 37926210

[66] Duda FP, Forte Neto FS, Fried E. 2023 Modelling of surface reactions and diffusion mediated by bulk diffusion. Phil. Trans. R. Soc. A **381**, 20220367. (10.1098/rsta.2022.0367) 37926211 PMC10645080

[67] Goldshtein V, Mariano PM, Mucci D, Segev R. 2023 Continuum kinematics with incompatible–compatible decomposition. Phil. Trans. R. Soc. A **381**, 20220372. (10.1098/rsta.2022.0372) 37926213

[68] Mariano PM. 2023 A certain counterpart in dissipative setting of the Noether theorem with no dissipation pseudo-potentials. Phil. Trans. R. Soc. A **381**, 20220375. (10.1098/rsta.2022.0375) 37926214

[69] Markenscoff X. 2023 Loss of pseudo-momentum, energy-release rate and the effective mass of a moving dislocation. Phil. Trans. R. Soc. A **381**, 20220232. (10.1098/rsta.2022.0232) PMC10876063 37926205

[70] Mucci D, Saracco A. 2023 Weak elastic energy of irregular curves. Phil. Trans. R. Soc. A **381**, 20220370. (10.1098/rsta.2022.0370) 37926207

[71] Pradhan SP, Yavari A. 2023 Accretion–ablation mechanics. Phil. Trans. R. Soc. A **381**, 20220373. (10.1098/rsta.2022.0373) 37926212

[72] Ruggeri T. 2023 Eckart equations, Maxwellian iteration and relativistic causal theories of divergence type. Phil. Trans. R. Soc. A **381**, 20220371. (10.1098/rsta.2022.0371) 37926206

